# Isolated Tillaux Fracture in a Skeletally-Mature Patient: A Rare Presentation

**DOI:** 10.7759/cureus.36910

**Published:** 2023-03-30

**Authors:** G. R. Joshi, Anish Tawde, Aditya Seth, Isha Seth, Ram K Aiyappan, Sunayana Singh, Muhammad Naqvi, Anikrit Shrivastava, Gaurav K Agrawal, J. S. S. N. Manohar

**Affiliations:** 1 Orthopedics, Bharati Vidyapeeth Medical College, Pune, IND; 2 Orthopedics and Traumatology, KIMS (Krishna Institute of Medical Sciences) Sunshine Hospital, Hyderabad, IND; 3 Obstetrics and Gynecology, Amrita Hospital, Faridabad, IND; 4 General Surgery, Amrita Hospital, Faridabad, IND; 5 Obstetrics and Gynecology, Government Medical College and Hospital, Chandigarh, IND; 6 Orthopedics, Pandit Bhagwat Dayal Sharma Post Graduate Institute of Medical Sciences, Rohtak, IND; 7 Orthopedics and Traumatology, Bharati Vidyapeeth Medical College, Pune, IND

**Keywords:** rare, early mobilization, open reduction and internal fixation, ankle injury, isolated tillaux fracture

## Abstract

Isolated Tillaux fractures are uncommon injuries that occur due to external rotational forces acting on the ankle joint. They are more commonly seen in adolescents due to the presence of open epiphyses. In adults, isolated Tillaux fractures present as plafond fractures well described with the AO (Arbeitsgemeinschaft für Osteosynthesefragen) Classification as well as Types 1, 2, and 3 according to the degree of articular comminution present. They occur rarely and can be easily missed or misdiagnosed as other ankle injuries. A thorough clinical examination, combined with imaging studies such as X-rays and CT scans, can aid in accurate diagnosis and treatment planning. Management typically involves open reduction and internal fixation, followed by a short period of immobilization and early mobilization with non-weight bearing. We present a case report of a 27-year-old female who presented with an isolated Tillaux fracture of the ankle following a road traffic accident. This type of fracture is typically seen in teenagers and young adults due to the incomplete closure of the growth plate, which makes it more susceptible to injury. The patient underwent open reduction and internal fixation with a contoured three-hole 3.5 mm titanium T-plate, followed by immobilization in a plaster splint for one week. Early mobilization was encouraged with strict non-weight bearing for 8-10 weeks. Follow-up at 12 weeks revealed complete union at the fracture site, with the patient being asymptomatic except for minimal pain and mild restriction in dorsiflexion.

## Introduction

Fractures in the anterolateral aspect of the distal tibia are frequently caused by avulsion of the anterior tibial tubercle, due to anterior-inferior tibiofibular ligament failing to rupture during injury, resulting in a juvenile Tillaux fracture in adolescents (Salter-Harris type III fracture) and a Tillaux fracture in adults [1]. The classification of this type of adult Tillaux fracture is type A, and if accompanied by medial injury, it is classified as type B [2]. The first report of this injury was by Cooper in 1822 [3], followed by Tillaux in 1848 [4]. The mechanism of injury for this fracture pattern, according to previous studies, is external rotation of the foot relative to the tibia [5,6]. Although it is typically observed in pediatric and adolescent populations and classified as a Salter-Harris III fracture through the epiphysis, it has been rarely reported in older populations. While it is frequently observed in children aged 12 to 14 years [5,7,8], it has also been documented in young adults [9] and those who have reached skeletal maturity [1,10,11], potentially due to delayed ossification of the growth plate. The goal of this case report is to present a unique case of tibial plafond fracture in an older adult mimicking the fracture geometry seen in a Tillaux fracture, emphasizing the importance of its accurate diagnosis to avoid possible complications such as premature degenerative arthritis and restricted ankle mobility.

## Case presentation

**Figure 1:**
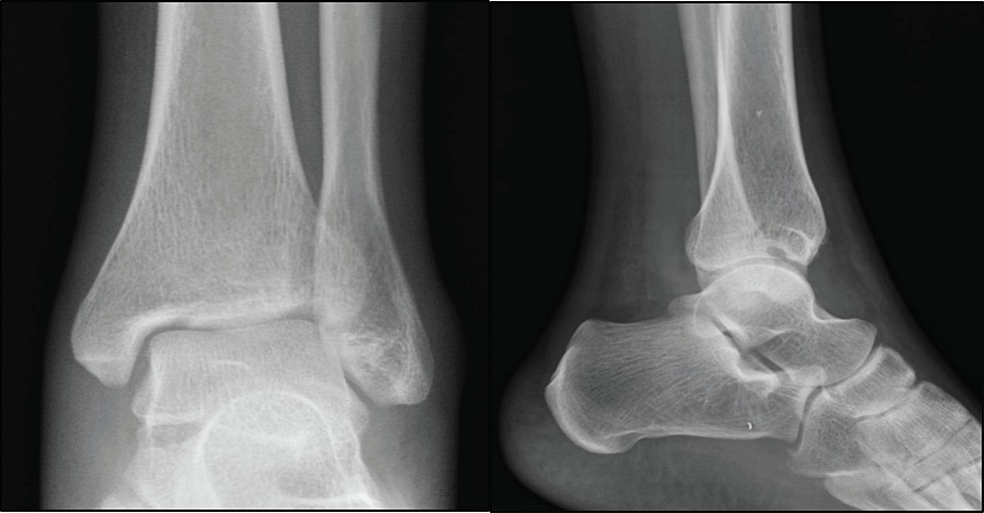
X-ray at presentation showing minimally displaced tibial plafond fracture

A 27-year-old female presented with pain, swelling, and restricted movements over her left ankle following a road traffic accident. On examination, moderate diffuse tenderness and swelling were noted over the anterior and anterolateral aspects of the left ankle with no distal neurovascular compromise. X-ray imaging was suggestive of a minimally displaced distal tibia plafond fracture (Figure 1).

**Figure 2:**
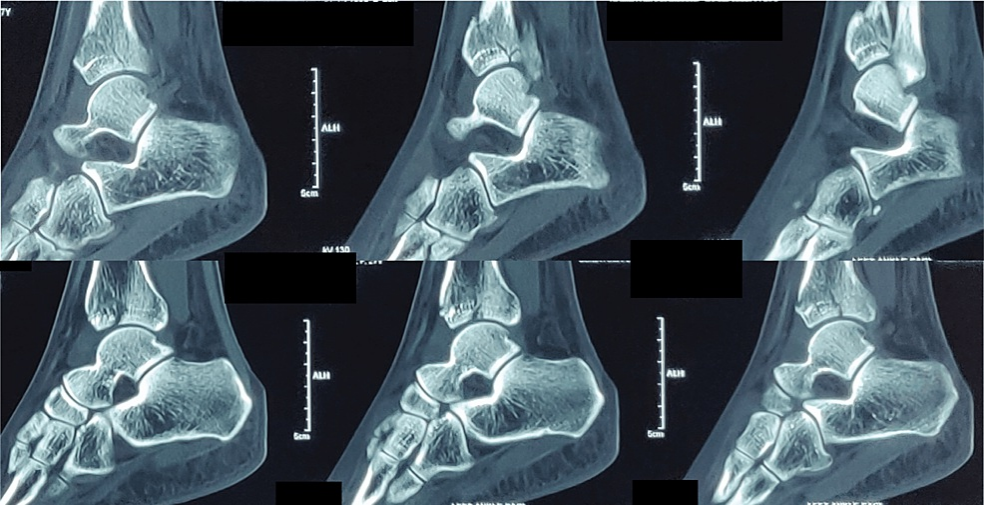
Saggital sections of CT scan of affected ankle

**Figure 3:**
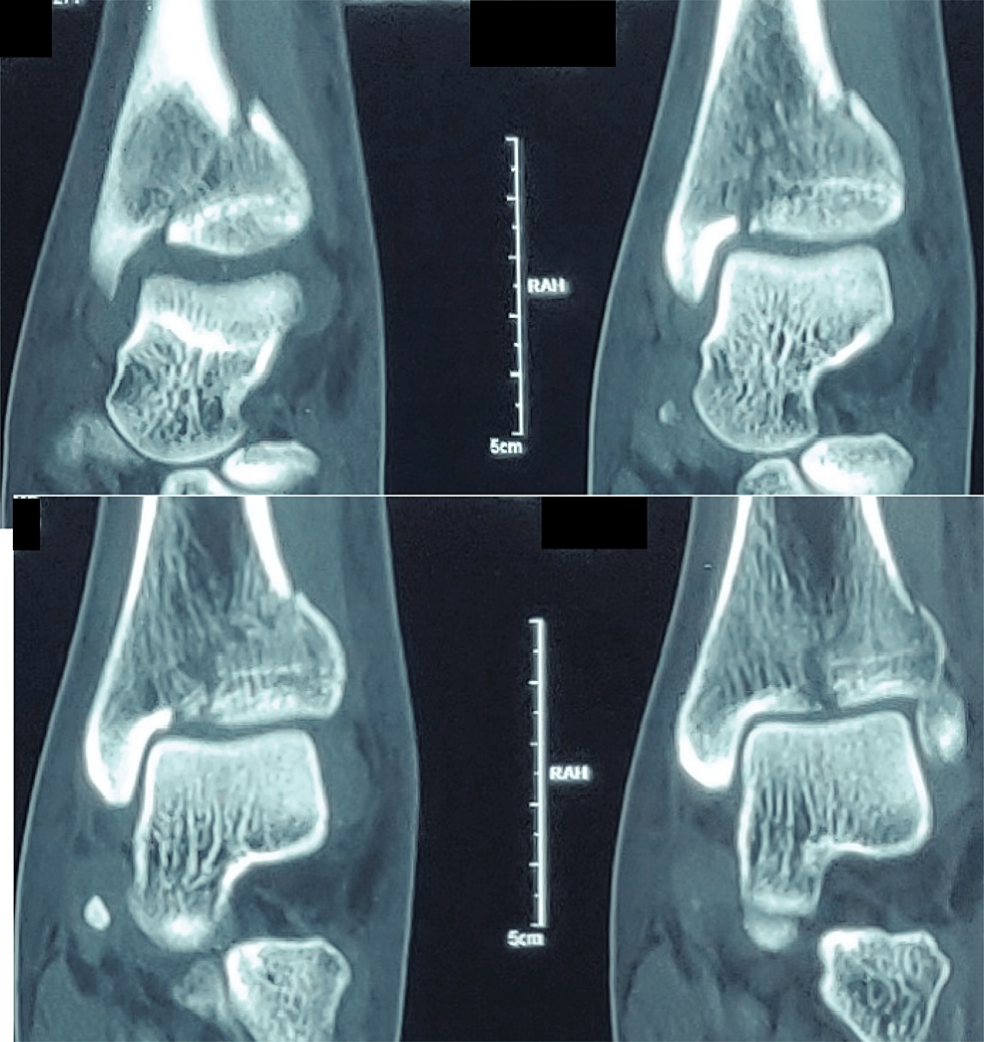
Coronal sections of CT scan of affected ankle

**Figure 4:**
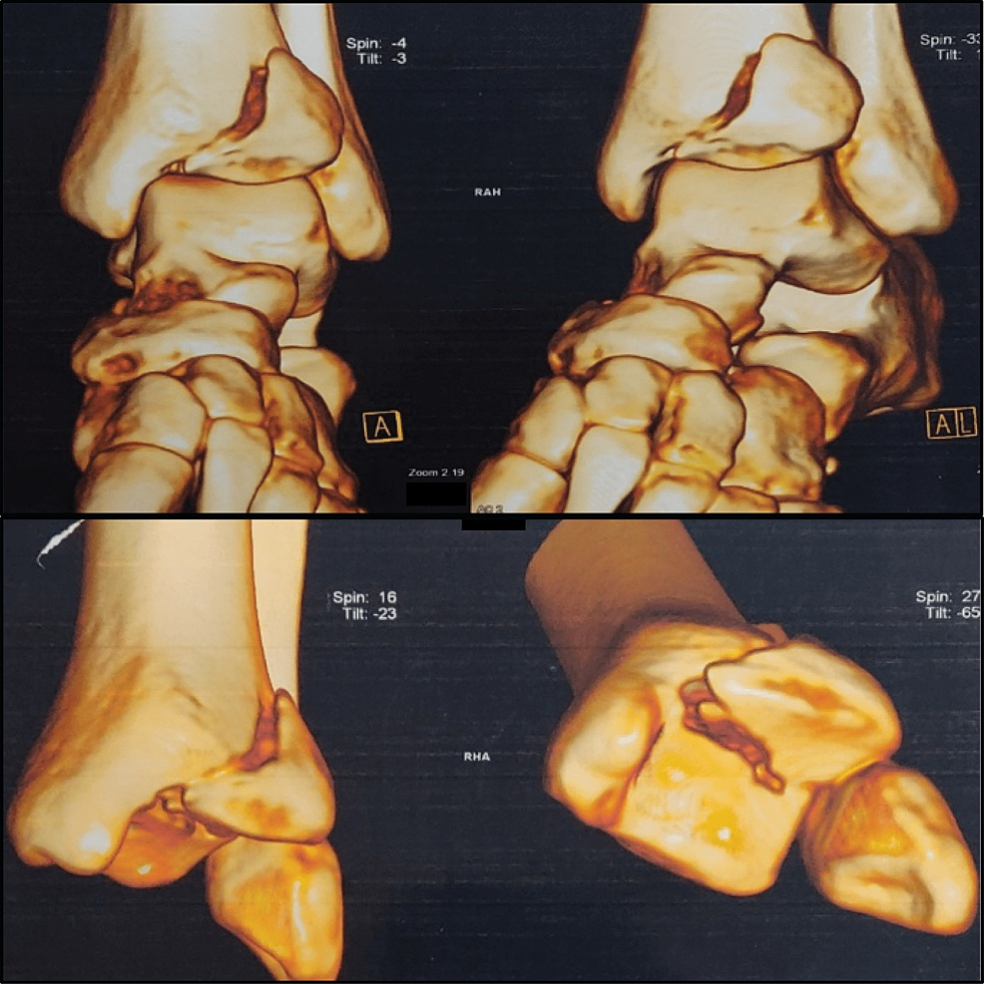
Image showing three-dimensional reconstruction of fracture

However, a CT scan with three-dimensional (3D) reconstruction ( Figures 2-4) was performed to further elucidate the fracture pattern, revealing approximately 40% involvement of the plafond with intra-articular extension.

**Figure 5:**
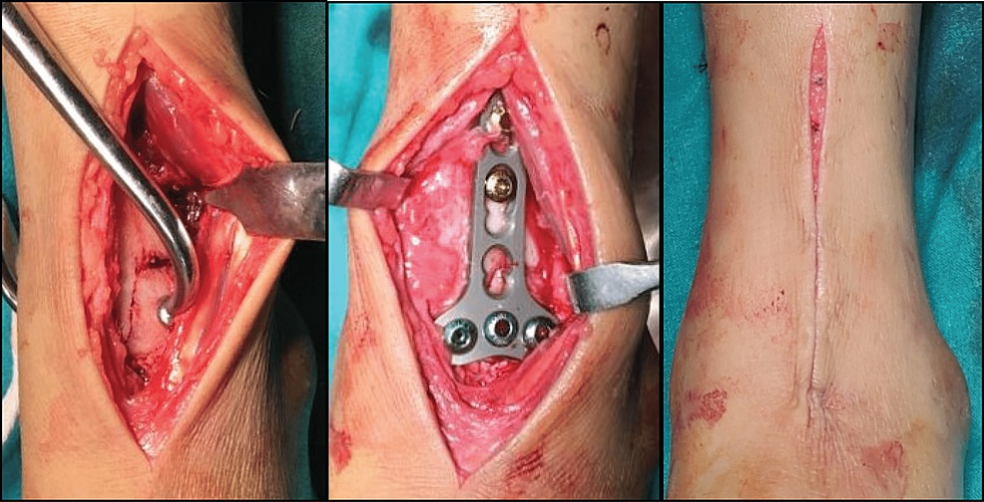
Intra-operative pictures showing the fracture fixed with distal radius T buttress plate

**Figure 6:**
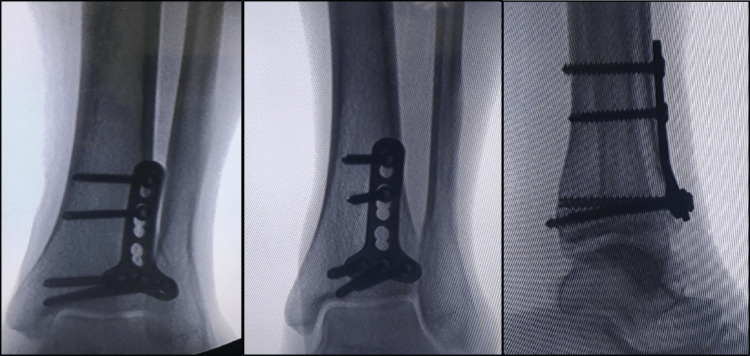
Intra-operative fluoroscopic images

**Figure 7:**
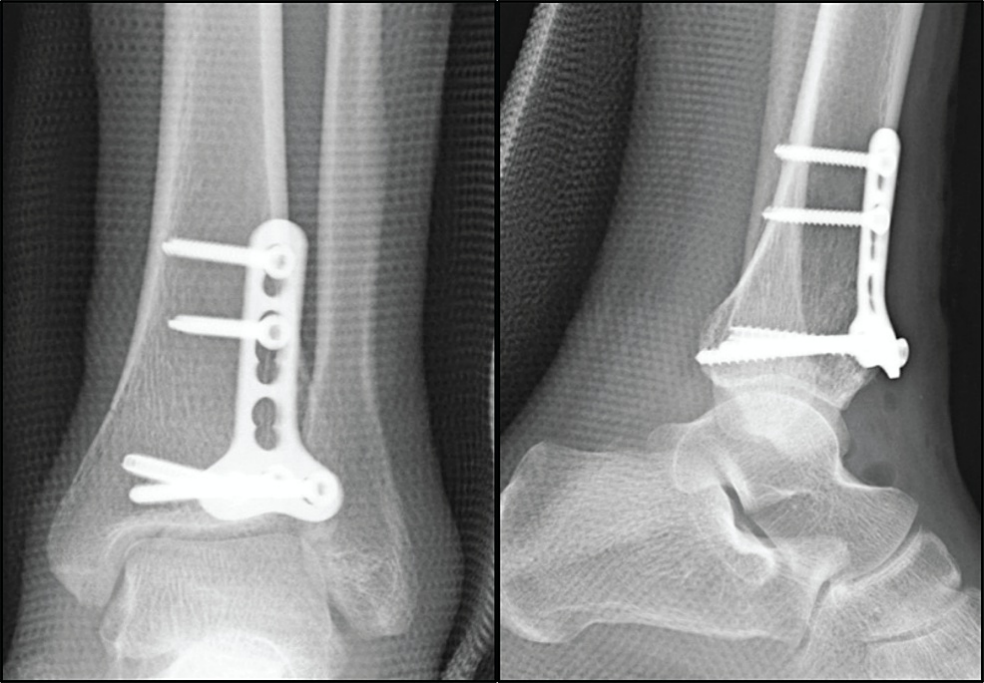
Postoperative radiograph showing the fixation and immobilization with plaster splint

Open reduction and internal fixation were performed through an anterolateral approach using a contoured three-hole 3.5 mm titanium T-plate (Figures 5-7).

**Figure 8:**
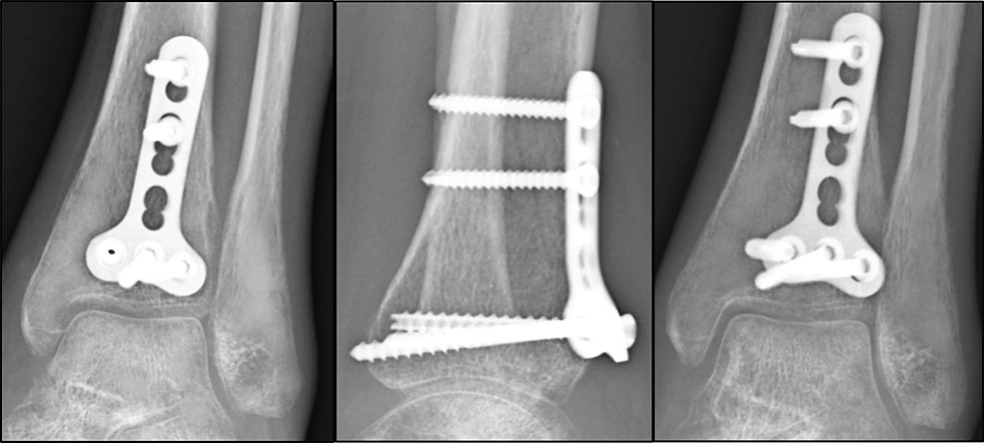
X-rays done at 12 weeks showing complete union at the fracture site

Postoperatively, the ankle was immobilized in a plaster splint for one week, with early mobilization encouraged and strict non-weight bearing for 8-10 weeks. The patient was followed up at two, six, and 12 weeks and was found to be asymptomatic at the end of 12 weeks with minimal pain and mild restriction in dorsiflexion. X-rays taken at 12 weeks showed complete union at the fracture site (Figure 8).

## Discussion

Avulsion fractures of the distal tibia are rare in adults, while they are more common in children due to the weaker epiphyseal plate in comparison to the ligamentous structures [6]. The incidence of Tillaux fractures in children ranges from approximately 5.2% to 10% [5,12], but the incidence of isolated Tillaux fractures in adults has not been quantified in current data.

The AO (Arbeitsgemeinschaft für Osteosynthesefragen) Classification system for fractures is a comprehensive system that takes into account various characteristics of the fracture, including the degree of displacement, the location of the fracture line, and the involvement of other structures such as ligaments. Since the term "Tillaux fracture" is limited to the pediatric and adolescent population having a partially open growth plate, the AO Classification system for distal tibia plafond fractures can be used for correlating such rare fracture patterns in an adult. In the AO Classification system for distal tibia plafond fractures, Tillaux fractures would be classified as AO 43B2 fractures. This classification refers to a Type B fracture (partial articular) that involves the lateral portion of the tibial plafond and extends into the epiphysis. The "2" designation indicates that the fracture is partially displaced. This classification can be further refined based on the level of displacement and the specific fracture pattern observed on imaging, and is used to guide treatment decisions and predict outcomes

If the fracture displacement measures less than 2 mm, non-surgical management is possible through immobilization using non-weight-bearing casts or ankle braces for six weeks. However, if the displacement of the fragment exceeds 2 mm, a CT scan may be necessary to accurately assess the fracture pattern and displacement. Subsequently, the close reduction under general anesthesia by axial traction and internal rotation of the foot, followed by immobilization in a cast with the foot in internal rotation, is commonly practiced. Nevertheless, this procedure may fail due to the presence of bony fragments, periosteum, or soft tissue interposed between the fracture fragment and tibia. If displacement exceeds 2 mm after attempted close reduction, surgical intervention such as open reduction and internal fixation is recommended to achieve anatomical alignment [13].

Recently, percutaneous fixation assisted with arthroscopy, a minimally invasive surgical technique, has been used to treat certain types of ankle fractures, including some plafond fractures and Tillaux fractures. This technique involves inserting small instruments and screws through small incisions in the skin, and using an arthroscope to visualize the inside of the joint and guide the placement of the screws. However, due to limitations of exposure in the field of foot and ankle arthroscopy as well as necessary equipment, it has not been widely practiced. 

In this case report, we presented a 27-year-old female who sustained a displaced distal tibia plafond fracture following a road traffic accident. The initial X-ray of the patient's ankle was suggestive of a minimally displaced fracture, but a CT scan revealed a 40% involvement of the plafond with an intra-articular extension (AO 43B2). With the advent of today's modern-day advanced imaging and technology, CT with 3D reconstruction has become imperative for diagnosis, assessment of geometry of intra-articular fractures, and preoperative surgical planning. Since the fracture was displaced, it warranted surgical treatment.

The postoperative management of distal tibia plafond AO 43B2 fractures, with characteristics similar to a Tillaux fracture, is crucial to achieve a successful outcome. Early mobilization of the ankle is important to prevent stiffness and promote joint motion, but weight-bearing should be avoided until radiographic evidence of union is observed.

## Conclusions

In conclusion, isolated Tillaux fracture in an adult are rare, falling under the subtype 43 B of AO Classification for distal tibial plafond fractures and require a comprehensive approach for optimal management. Surgical intervention with stable fixation and early mobilization is essential to achieve a successful outcome. A thorough follow-up is important to detect and manage any complications that may arise. Our case report demonstrates that the anterolateral approach with a contoured T-plate can provide stable fixation and allow for early mobilization, resulting in a successful outcome for the patient.
